# Depression, Anxiety and Emotional Problems Among Healthcare Workers

**DOI:** 10.3390/healthcare14213823

**Published:** 2026-11-09

**Authors:** Sai-fu Fung

**Affiliations:** Department of Social and Behavioural Sciences, City University of Hong Kong, Kowloon, Hong Kong; sffung@cityu.edu.hk

## 1. Introduction

The World Health Organization (WHO) has called attention to the mental health and well-being of health and care workers [1], noting that these factors critically affect patient care, raise public health concerns, and could contribute to a wider global crisis [2]. Similarly, at the 76th WMA General Assembly in Porto, Portugal (October 2025), the World Medical Association emphasized physicians’ well-being and outlined “key elements and deliverables of national occupational safety programmes that help promote physician well-being, decrease work-related risks and prevent occupational diseases in health care workers” [3]. The WHO reported that the situation worsened during the peak of the COVID-19 pandemic in 2022 [1], and the United States enacted the Dr. Lorna Breen Health Care Provider Protection Act to prevent suicide, burnout, mental health conditions, and substance use disorders among health workers. Despite these efforts, strikes by health and care workers driven by stress and burnout are not uncommon in many countries and have significantly disrupted health systems worldwide [4].

In this context, this Special Issue (SI) examines pressing mental health concerns among healthcare workers—including, but not limited to, depression, anxiety, burnout, and other emotional problems. The COVID-19 pandemic and ongoing global health challenges have intensified already-high stress levels in the profession, underscoring the need for comprehensive research on their psychological well-being. Healthcare workers often experience prolonged exposure to trauma, heavy workloads, and emotional exhaustion in strained work settings, increasing the risk of serious mental health problems.

This SI includes twelve studies—one systematic review (Contribution 9) and eleven original research articles (Contributions 1–8 and 10–12)—that document depression, anxiety, and related emotional problems among healthcare workers in multiple countries, including Australia (Contribution 2), Belgium (Contribution 6), Portugal (Contribution 3), Saudi Arabia (Contribution 1), Singapore (Contributions 11, 12), Spain (Contributions 2, 8, 10), and the United States (Contributions 5, 7).

## 2. Overview of Contributions

This SI aims to stimulate rigorous, interdisciplinary discussion of pressing issues affecting healthcare workers, including stress, well-being, patient-care quality, burnout, emotional symptoms, anxiety, trauma, quality of life, and financial strain. Contributing authors fostered a rigorous interdisciplinary discussion of pressing issues affecting healthcare workers—including stress, well-being, patient-care quality, burnout, emotional symptoms, anxiety, trauma, quality of life, psychological distress, and financial strain—across professions such as physicians, nurses, pharmacists, physiotherapists, hospital support staff, health sciences faculty, nursing students, caregivers, and frontline teams (e.g., ED and maternity unit staff) (Contributions 1–2, 6–7, 10, 12). This Special Issue also features new intervention strategies and programmes supported by empirical evidence that improve resilience, team connectedness, job confidence, well-being, happiness, meaningfulness at work, mood, stress reduction, and distress management (Contributions 3–5, 8, 11, 12). Finally, it includes a systematic review evaluating the validity of key outcome measures used in empirical studies—such as tools for assessing depression, anxiety, stress, and burnout (Contribution 9).

Ensuring that study measures are valid and reliable is essential for programme evaluation and research. Contribution 9 used a narrative integrative review of 35 articles examining three widely used instruments—the Depression, Anxiety and Stress Scales (DASS), the Maslach Burnout Inventory (MBI), and the Zung Self-Rating Depression Scale (Zung SDS)—to assess depression, anxiety, stress, and burnout in healthcare workers. Its findings guide practitioners and researchers on the suitability of these scales in different contexts.

Two Spain-based studies examined challenges faced by healthcare workers and informal caregivers. Contribution 10 surveyed 253 health-sciences university faculty to assess perceived stress, burnout, and reduced professional quality of life stemming from their dual roles as educators and clinicians. Contribution 2 studied 344 informal caregivers supporting dependent family members and used structural equation modelling (SEM) to explore how caregiver burden and stress relate to anxiety and depression, providing empirical evidence of the importance of support for caregivers’ mental health.

Compassion fatigue, conceptualized as emotional and physical exhaustion arising from caregiving relationships, was examined qualitatively in Contribution 6. Interviews with 10 experienced maternity professionals (midwives and gynecologists with 6–30 years’ experience) indicated the need for multilevel responses (individual, team, and systemic) to address these issues in maternity care.

Contribution 1 introduced an innovative analytic approach—fuzzy association rule mining (FARM)—applied to data from 204 healthcare workers to identify factors associated with work-related stress and anxiety under high-risk conditions such as the COVID-19 pandemic. FARM can complement methods like SEM by uncovering complex or non-linear associations that standardized-weight approaches may miss.

Contribution 7 reported on 351 Emergency Department staff in several U.S. hospitals. Burnout was reported by 31.7% of respondents and psychiatric symptoms by 20.1%. The reported figures warrant close attention, as 75% of those with symptoms were reluctant to seek formal psychological support because of practical and emotional barriers, highlighting an urgent need for targeted interventions to reduce such barriers.

Together, Contributions 11 and 12 demonstrate complementary approaches to protecting healthcare workers’ mental health in Singapore. Contribution 11 evaluated a staff well-being engagement programme at a Singapore hospital built on three pillars: individual empowerment, leadership stewardship, and institutional support. Data from 314 participants plus qualitative feedback showed improved happiness and meaningfulness in clinical work, but ongoing challenges remain around organizational recognition, management responsiveness and transparency, and psychological safety. Contribution 12 examined demographic, clinical, and lifestyle factors to assess psychological distress among 1260 staff before (2018–2022) and after (2023–2025) the pandemic at Singapore’s National University Polyclinics, a public primary-care network that provides acute care, chronic disease management, women’s and children’s services, and dental care. The study provides an important model for other countries’ healthcare systems to monitor staff mental well-being regularly, offer timely support, and ultimately enhance patient safety and quality of care.

This SI also presents several intervention studies. Contribution 3 piloted a virtual reality-based relaxation session for frontline workers in Portugal who served during the acute COVID-19 phase; Contribution 5 evaluated a digital mental health intervention (SigBee^®^, Austin, TX, USA) for disaster service providers in the United States; Contribution 4 tested an 8-week yoga programme for Australian nursing students to enhance stress management and resilience; and Contribution 8 assessed a psychoeducational emotional-well-being programme for healthcare workers in Spain; findings from five focus groups (*n* = 38) indicated participant benefits but also the need for longer-term, individual-focused strategies and system-level support.

## 3. Future Priorities

Evaluating outcome measures is essential for assessing future interventions and programmes aimed at improving the well-being of healthcare workers and related stakeholders. Standard psychometric procedures employing Classical Test Theory (CTT) or Item Response Theory (IRT) are commonly used to validate these scales. Over time, shorter, easy-to-administer versions of instruments become important to facilitate programme evaluation and research in busy clinical settings.

Advances in technology may also improve frontline working environments—for example, using robotic or humanoid assistance for surgical tasks. Robotic platforms such as the da Vinci Surgical System or Sentire Surgical System designed for minimally invasive procedures can reduce post-operative complications [5,6,7] and are being further developed with AI enhancements to expand their capabilities [8]. These technologies have the potential to reduce work-related stress for healthcare workers and to improve patient satisfaction [9].

This Special Issue also generates several implications for government officials and senior health-system managers, particularly around governance to improve healthcare workers’ well-being. Key actionable recommendations include: (i) introducing workplace safeguards [1,10]; (ii) adopting staff well-being as a core governance metric [11]; (iii) investing in psychological safety and a just culture [11,12] (see also Contributions 11 and 12); (iv) embedding flexibility and workload management into policy [11,13,14]; and (v) strengthening leadership development from senior management downward to address issues such as management responsiveness and transparency highlighted in Contribution 11 [15]. Over the long term, multilevel systems—government bodies and health-system leadership—should adopt routine preventive checks and other proactive measures (Contribution 12). Preventive action is preferable to reactive, post-crisis responses when addressing stress, burnout, and other mental-health challenges among healthcare workers.

Methodological implications include the utility of the actor–partner interdependence model (APIM), a framework for analyzing dyadic data that separates actor effects (how an individual’s predictor affects their own outcome) from partner effects (how an individual’s predictor affects the other person’s outcome) [16,17]. APIM offers researchers an additional approach to evaluate interventions in healthcare dyads—such as caregiver–dependent or survivor pairs (Contribution 7), doctor-patient relationships [18], or other closely connected pairs. It can be implemented via regression-based multilevel models or structural equation modelling and applied in both cross-sectional and longitudinal designs [19].

## 4. Conclusions

This Special Issue serves as a starting point for further work on addressing the wide range of psychosocial challenges faced by healthcare workers in diverse settings. Much remains to be done, particularly adopting a multilevel approach that involves the international community, governments, and relevant stakeholders. Technology can support this effort, but we also need more innovative interventions and stronger research methods and data-analysis techniques. Sharing best practices across institutions and countries will help improve healthcare workers’ well-being. Above all, this collection is a call for greater attention to the often-unseen heroes who risked their lives to care for others during the pandemic and continue to do so in the face of global health challenges.
